# Bioinformatics identification of crucial genes and pathways associated with hepatocellular carcinoma

**DOI:** 10.1042/BSR20181441

**Published:** 2018-11-09

**Authors:** Xueren Gao, Xixi Wang, Shulong Zhang

**Affiliations:** 1 Department of Pediatric Endocrinology and Genetics, Shanghai Institute for Pediatric Research, Xinhua Hospital, School of Medicine, Shanghai Jiao Tong University, Shanghai 200092, China; 2 Department of Women’s Health Care, Maternal and Child Health Care Hospital of Jiangyin, Maternal and Child Health Care Family Planning Service Center of Jiangyin, Wuxi 214000, China; 3 Department of General Surgery, Xuhui District Central Hospital of Shanghai, Shanghai 200031, China

**Keywords:** hepatocellular carcinoma, Crucial gene, Pathway, Survival analysis

## Abstract

Hepatocellular carcinoma (HCC) is a major cause of cancer-related death worldwide. Up to date, HCC pathogenesis has not been fully understood. The aim of the present study was to identify crucial genes and pathways associated with HCC by bioinformatics methods. The differentially expressed genes (DEGs) between 14 HCC tissues and corresponding non-cancerous tissues were identified using limma package. Gene Ontology (GO) and KEGG pathway enrichment analysis of DEGs were performed by clusterProfiler package. The protein–protein interaction (PPI) network of DEGs was constructed and visualized by STRING database and Cytoscape software, respectively. The crucial genes in PPI network were identified using a Cytoscape plugin, CytoNCA. Furthermore, the effect of the expression level of the crucial genes on HCC patient survival was analyzed by an interactive web-portal, UALCAN. A total of 870 DEGs including 237 up-regulated and 633 down-regulated genes were identified in HCC tissues. KEGG pathway analysis revealed that DEGs were mainly enriched in complement and coagulation cascades pathway, chemical carcinogenesis pathway, retinol metabolism pathway, fatty acid degradation pathway, and valine, leucine and isoleucine degradation pathway. PPI network analysis showed that *CDK1, CCNB1, CCNB2, MAD2L1, ACACB, IGF1, TOP2A*, and *EHHADH* were crucial genes. Survival analysis suggested that the high expression of *CDK1, CCNB1, CCNB2, MAD2L1*, and *TOP2A* significantly decreased the survival probability of HCC patients. In conclusion, the identification of the above crucial genes and pathways will not only contribute to elucidating the pathogenesis of HCC, but also provide prognostic markers and therapeutic targets for HCC.

## Introduction

Hepatocellular carcinoma (HCC) is the most frequent primary liver malignancy and a major cause of cancer-related death worldwide, especially in developing countries [1]. The mean survival time of HCC patients without intervention is estimated between 6 and 20 months. Although surgical resection, orthotopic liver transplantation, radiofrequency thermal ablation and sorafenib have been used to treat HCC, treatment outcomes are still unsatisfactory due to postsurgical recurrence and drug resistance [2–4]. Therefore, a further investigation into the underlying mechanisms of HCC initiation and progression is urgently needed, which will contribute to the discovery of novel diagnostic and therapeutic targets for HCC.

In the past decades, there have been many reports of genes and pathways involved in HCC initiation and progression [5–8]. For instances, Villanueva et al. [5] used genome-wide methylation profiling not only to discover many genes that are aberrantly methylated in HCC, such as *RSSFA1, IGF2, APC, RASSF5, SFRP5, NEFH, SEPT9, EFNB2* and *FGF6*, but also to point to signaling pathways clearly deregulated by DNA methylation in HCC, such as IGF, PI3K, TGF-b, and WNT signaling pathways. Zhao et al. [6] found that methylation-induced ASPP1 and ASPP2 silence promoted tumor growth in HCC, which might serve as potential treatment targets. Wu et al. [7] observed that the expression level of OCIAD2 in the tumor tissues was much lower than that in the corresponding adjacent normal tissues, and OCIAD2 suppressed tumor growth and invasion via AKT pathway in HCC. Lee et al. [8] found that TonEBP promoted hepatocellular carcinogenesis, recurrence and metastasis, and targeting TonEBP may be an attractive strategy to prevent recurrence as well as hepatocarcinogenesis and metastasis.

Although an increasing number of studies aimed to clarify the molecular mechanism of HCC initiation and progression, the progress of the relevant studies is not obvious. Gene expression microarray technology has been used to simultaneously detect the expression level of thousands of genes in cells and tissues, which helps researchers discover the crucial genes and pathways associated with disease. In the present study, we utilized this high-throughput technology to screen differential expression genes (DEGs) between HCC tissues and corresponding non-cancerous tissues, and then identified the crucial genes and pathways associated with HCC by bioinformatics methods. In addition, the effect of the expression level of the crucial genes on HCC patient survival was evaluated by UALCAN (http://ualcan.path.uab.edu) [9].

## Materials and methods

### Microarray data

The raw microarray data of GSE84402 including 14 HCC tissues and corresponding non-cancerous tissues were obtained from the Gene Expression Omnibus (GEO) database (http://www.ncbi.nlm.nih.gov/geo/). These data were based on GPL570 platform (Affymetrix Human Genome U133 Plus 2.0 Array) and contributed by Wang et al [10].

Data preprocessing was performed using the affy package V1.56.0 and the Robust Multichip Averaging (RMA) algorithm in R V3.4.4 [11]. The probeset IDs were converted into the corresponding gene symbol using the annotation information derived from platform GPL570. If multiple probe sets correspond to one gene, the mean expression values of those probesets were obtained.

### Identification of DEGs

The limma package V3.34.9 in R was used to identify DEGs in HCC tissues compared with corresponding non-cancerous tissues [12]. The *t* test and Benjamini–Hochberg method were used to calculate the *P*-value and FDR, respectively. The DEGs were screened out according to adjusted *P*-value <0.05 and | logFC | ≥1.

### Gene Ontology (GO) and pathway enrichment analysis of DEGs

The clusterProfiler V3.6.0 is an ontology-based R package that not only automates the process of biological-term classification and the enrichment analysis of gene clusters, but also provides a visualization module for displaying analysis results [13]. In the present study, the clusterProfiler package was used to identify and visualize the GO terms and KEGG pathways enriched by DEGs. *P*-value <10^−6^ was set as the cut-off criterion for the significant enrichment.

### Analysis of the protein–protein interaction (PPI) network of DEGs

STRING database (www.string-db.org) collected and integrated known and predicted protein–protein association data for a large number of organisms, including *Homo sapiens* [14]. In the present study, STRING was used to construct the PPI network of DEGs with minimum required interaction score 0.7. Cytoscape software V3.5.1 was used to display the PPI network [15]. CytoNCA V2.1.6 was a cytoscape plugin for centrality analysis of protein interaction networks, which could be utilized to identify crucial nodes (genes) in the network [16]. In the present study, the crucial genes were identified based on four different centrality measures, including eigenvector centrality (EGC), degree centrality (DC), betweenness centrality (BC), and closeness centrality (CC). According to the centrality values of genes in the PPI network, the top 3 ranked genes were identified as the crucial genes.

### The effect of the expression level of the crucial genes on HCC patient survival

UALCAN is a user-friendly, interactive web resource for analyzing cancer transcriptome data from The Cancer Genome Atlas (TCGA). It uses TCGA level 3 RNA-seq and clinical data from 31 cancer types to carry out the following work: (a) analyze relative expression of genes across tumor and normal samples, as well as in various tumor sub-groups based on individual cancer stages, tumor grade, race, body weight, or other clinicopathologic features; (b) assess the effect of gene expression level and clinicopathologic features on patient survival; and (c) identify the top up- and down-regulated genes in individual cancer types. In the present study, it was utilized not only to validate the expression level of the crucial genes, but also to estimate the effect of the expression level of the crucial genes on HCC patient survival by Kaplan–Meier curve and Log-rank test.

## Results

### Identification of DEGs

**Figure 1:**
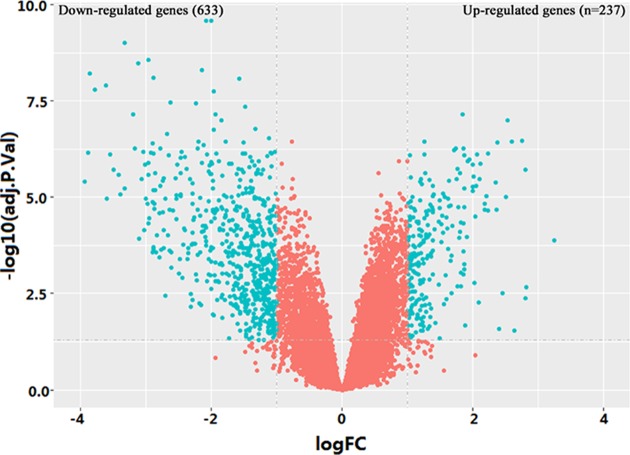
Volcano plot of microarray data (upper-left and upper-right blue dots stand for down- and up-regulated genes in HCC, respectively

A total of 870 DEGs were identified, including 237 up-regulated genes and 633 down-regulated genes in HCC tissues (Figure 1). The top 10 up-regulated DEGs were *GPC3, CTHRC1, GINS1, PEG10, TOP2A, SPINK1, CDKN3, CCNB1, RRM2*, and *TRIM71*. The top 10 down-regulated DEGs were *SLC22A1, MT1M, GYS2, OIT3, FCN3, APOF, LINC00844, GBA3, CRHBP*, and *CYP1A2* (Table 1).

**Table 1:** The top 10 up- and down-regulated genes in HCC

Gene symbol	logFC	adj.P.Val	Expression	Gene symbol	logFC	adj.P.Val	Expression
GPC3	3.24	0.00013	up	SLC22A1	-4.14	2.60E-06	down
CTHRC1	2.82	0.0021	up	MT1M	-3.93	3.94E-06	down
GINS1	2.80	1.89E-06	up	GYS2	-3.87	7.10E-07	down
PEG10	2.80	0.0042	up	OIT3	-3.85	6.20E-09	down
TOP2A	2.75	3.50E-07	up	FCN3	-3.77	1.60E-08	down
SPINK1	2.63	0.028	up	APOF	-3.60	1.23E-08	down
CDKN3	2.60	3.64E-07	up	LINC00844	-3.59	1.10E-05	down
CCNB1	2.53	1.01E-07	up	GBA3	-3.54	7.87E-07	down
RRM2	2.50	1.01E-05	up	CRHBP	-3.49	1.93E-06	down
TRIM71	2.45	0.0030	up	CYP1A2	-3.41	2.68E-06	down

### GO and KEGG pathway enrichment analysis of DEGs

**Figure 2:**
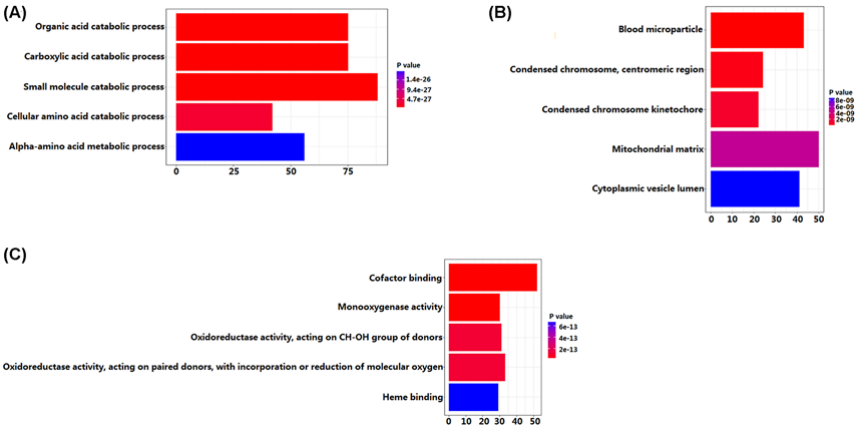
The top 5 GO terms enriched by DEGs (**A**, biological process; **B**, cellular component; **C**, molecule function)

**Figure 3:**
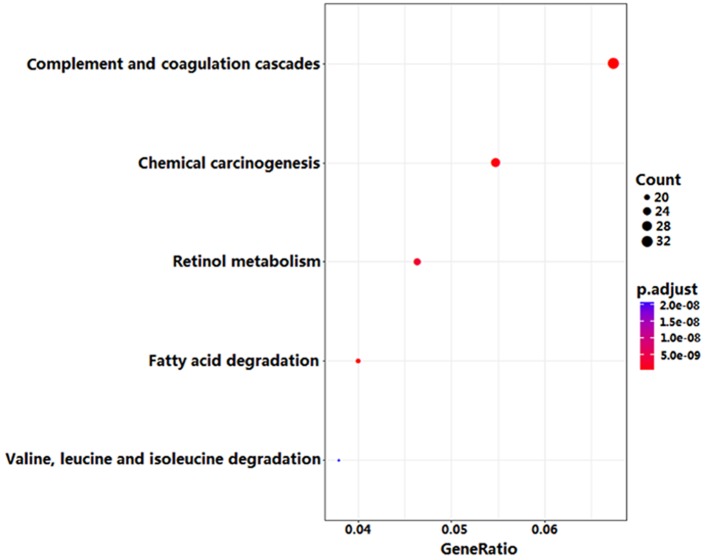
The top 5 KEGG pathways enriched by DEGs

GO enrichment analysis showed that DEGs were significantly enriched in 79 biological processes (BPs), 17 molecule functions (MFs) and 9 cellular components (CCs). The top 5 BPs included ‘organic acid catabolic process’, ‘carboxylic acid catabolic process’, ‘small molecule catabolic process’, ‘cellular amino acid catabolic process’, and ‘alpha-amino acid metabolic process’. The top 5 MFs included ‘cofactor binding’, ‘monooxygenase activity’, ‘oxidoreductase activity, acting on CH-OH group of donors’, ‘oxidoreductase activity, acting on paired donors, with incorporation or reduction of molecular oxygen’, and ‘heme binding’. The top 5 CCs included ‘blood microparticle’, ‘condensed chromosome, centromeric region’, ‘condensed chromosome kinetochore’, ‘mitochondrial matrix’, and ‘cytoplasmic vesicle lumen’ (Figure 2). Furthermore, KEGG pathway enrichment analysis indicated that DEGs were significantly enriched in 11 pathways. The top 5 pathways included ‘complement and coagulation cascades pathway’, ‘chemical carcinogenesis pathway’, ‘retinol metabolism pathway’, ‘fatty acid degradation pathway’ and ‘valine, leucine and isoleucine degradation pathway’ (Figure 3).

### PPI network of DEGs

**Figure 4:**
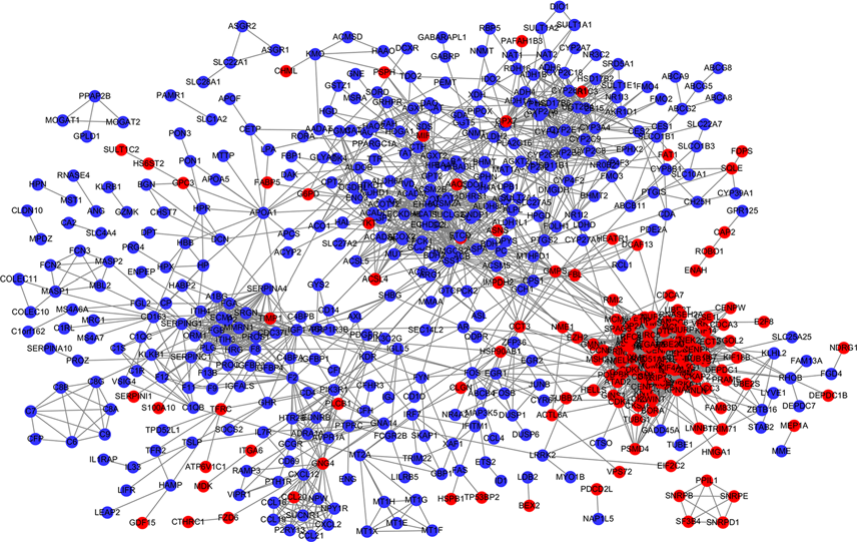
PPI network plot of DEGs (red and blue nodes stand for up- and down- regulated genes in HCC, respectively)

The PPI network of DEGs were constructed, including 556 nodes (genes) and 3570 edges (interactions) (Figure 4). The centrality analysis of nodes in the PPI network showed that *CDK1, CCNB1, CCNB2, MAD2L1*, ACACB, *IGF1, TOP2A*, and *EHHADH* were crucial genes (Table 2). Therein the expression levels of *CDK1, CCNB1, CCNB2, MAD2L1*, and *TOP2A* were up-regulated in HCC. The expression levels of *ACACB, IGF1*, and *EHHADH* were down-regulated in HCC.

**Table 2:** The top 3 genes ranked by the node centrality of the PPI network

Rank	Eigenvector centrality		Degree centrality		Betweenness centrality		Closeness centrality	
	Gene symbol	Expression in HCC	Gene symbol	Expression in HCC	Gene symbol	Expression in HCC	Gene symbol	Expression in HCC
1	CDK1	up-regulated	CDK1	up-regulated	ACACB	down-regulated	ACACB	down-regulated
2	CCNB1	up-regulated	MAD2L1	up-regulated	IGF1	down-regulated	TOP2A	up-regulated
3	CCNB2	up-regulated	CCNB1	up-regulated	EHHADH	down-regulated	EHHADH	down-regulated

### Validation of the expression level of the crucial genes

**Figure 5:**
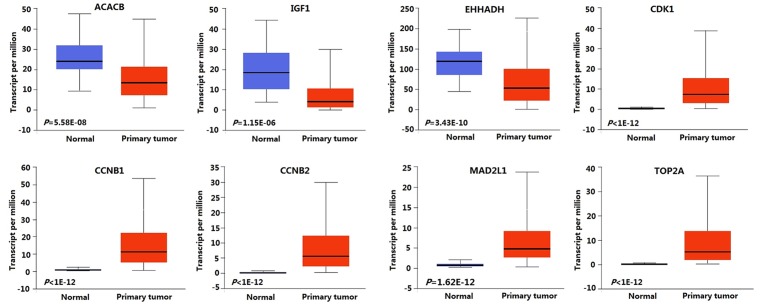
Boxplots showing the expression level of crucial genes in normal and HCC samples

UALCAN was used to validate the expression level of the crucial genes. The results showed that *ACACB, IGF1*, and *EHHADH* were significantly down-regulated in HCC, and *CDK1, CCNB1, CCNB2, MAD2L1*, and *TOP2A* were significantly up-regulated in HCC, which was consistent with the microarray results (Figure 5).

### The effect of the expression level of the crucial genes on HCC patient survival

**Figure 6:**
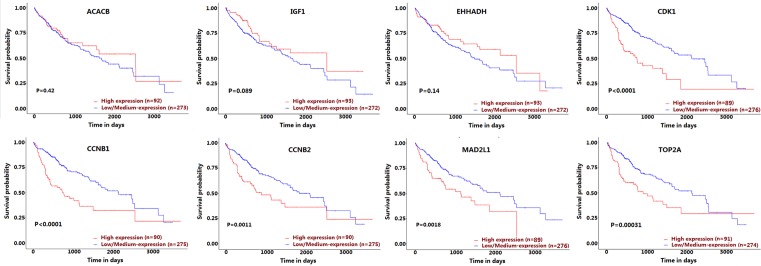
Kaplan–Meier plots showing the association of the expression level of crucial genes with HCC patient survival

Survival analysis based on TCGA data showed that the high expression of *CDK1, CCNB1, CCNB2, MAD2L1*, and *TOP2A* genes significantly decreased the survival probability of HCC patients (Figure 6).

## Discussion

In the present study, we explored the crucial genes and pathway associated with HCC by bioinformatics methods. By comparing gene expression profiles between 14 HCC tissues and corresponding non-cancerous tissues, we found 870 DEGs, including 237 up-regulated genes and 633 down-regulated genes in HCC tissues. Subsequently, GO and KEGG pathway enrichment analysis were performed to understand the biological functions of DEGs and pathways associated with HCC. GO analysis showed that DEGs were mainly involved in ‘organic acid catabolic process’, ‘carboxylic acid catabolic process’, ‘small molecule catabolic process’, ‘cellular amino acid catabolic process’, ‘alpha-amino acid metabolic process’, ‘cofactor binding’, ‘monooxygenase activity’, ‘oxidoreductase activity, acting on CH-OH group of donors’, ‘oxidoreductase activity, acting on paired donors, with incorporation or reduction of molecular oxygen’, and ‘heme binding’. KEGG pathway analysis showed that DEGs were mainly enriched in the following five pathways: ‘complement and coagulation cascades pathway’, ‘chemical carcinogenesis pathway’, ‘retinol metabolism pathway’, ‘fatty acid degradation pathway’, and “valine, leucine and isoleucine degradation pathway’.

According to the centrality of nodes in the PPI network, we identified the crucial DEGs, including *CDK1, CCNB1, CCNB2, MAD2L1*, ACACB, *IGF1, TOP2A*, and *EHHADH.* Subsequently, the expression level of the crucial genes was further validated based on *TCGA* data*. ACACB, IGF1*, and *EHHADH* were significantly down-regulated in HCC, and *CDK1, CCNB1, CCNB2, MAD2L1*, and *TOP2A* were significantly up-regulated in HCC, which was consistent with the microarray results. In these crucial genes, *CDK1, CCNB1, CCNB2, MAD2L1, IGF1, TOP2A*, and *EHHADH* had also been reported to be associated with HCC initiation and progression. Zhao et al. [17] found that CDK1 played an important role in the regulation of apoptin-induced apoptosis of HCC cells. Gao et al. [18] demonstrated that KPNA2 could accelerate cell cycle progression by up-regulating the expression of CCNB2 and CDK1 in HCC. Sun et al. [19] confirmed that the overexpression of p42.3 could up-regulate the expression of PCNA, CCNB1 and MAD2L1, and promote cell growth and tumorigenicity in HCC. Tang et al. [20] found that CAV1 conferred resistance of hepatoma cells to anoikis by activating IGF-1 pathway, providing a potential therapeutic target for HCC metastasis. Wong et al. [21] found that TOP2A overexpression in HCC correlated with early age onset, shorter patients survival, and chemoresistance. Suto et al. [22] observed decreased expression of EHHADH in HCC by the immunohistochemical staining technique. Although the role of ACACB in HCC was still not reported, previous studies indicated that ACACB played an important role in the initiation and progression of other cancers [23–25]. For instance, Jeon et al. [23] found that knockdown of ACACB could compensate for AMPK activation and facilitate anchorage-independent growth and solid tumor formation *in vivo*, whereas the activation of ACACB could attenuate these processes. Ho et al. [24] observed that the expression level of ACACB was significantly different between osteosarcoma and normal bone samples. The above findings suggested that *ACACB* might be a novel gene associated with HCC. To explore prognostic biomarkers for HCC, we utilized UALCAN to analyze the effect of the expression level of the crucial genes on HCC patient survival, and found that the high expressions of *CDK1, CCNB1, CCNB2, MAD2L1*, and *TOP2A* were associated with poor survival of HCC patients.

Taken together, our study finds the crucial genes and pathways associated with HCC, which will not only contribute to elucidating the pathogenesis of HCC, but also provide prognostic markers and therapeutic targets for HCC.

## Abbreviations

DEG: differentially expressed gene
HCC: hepatocellular carcinoma
PPI: protein–protein interaction
